# Quantification of Blood Flow Velocity in the Human Conjunctival Microvessels Using Deep Learning-Based Stabilization Algorithm

**DOI:** 10.3390/s21093224

**Published:** 2021-05-06

**Authors:** Hang-Chan Jo, Hyeonwoo Jeong, Junhyuk Lee, Kyung-Sun Na, Dae-Yu Kim

**Affiliations:** 1 Department of Electrical and Computer Engineering, Inha University, Incheon 22212, Korea; hjofficial@inha.edu (H.-C.J.); dydrl9713@inha.edu (H.J.); jhsky0919@naver.com (J.L.); 2 Center for Sensor Systems, Inha University, Incheon 22212, Korea; 3 Department of Ophthalmology & Visual Science, Yeouido St. Mary’s Hospital, College of Medicine, The Catholic University of Korea, Seoul 07345, Korea; 4 Inha Research Institute for Aerospace Medicine, Inha University, Incheon 22212, Korea

**Keywords:** blood flow velocity quantification, conjunctival microvessel, deep learning, image processing, motion correction, optical imaging system, vessel segmentation

## Abstract

The quantification of blood flow velocity in the human conjunctiva is clinically essential for assessing microvascular hemodynamics. Since the conjunctival microvessel is imaged in several seconds, eye motion during image acquisition causes motion artifacts limiting the accuracy of image segmentation performance and measurement of the blood flow velocity. In this paper, we introduce a novel customized optical imaging system for human conjunctiva with deep learning-based segmentation and motion correction. The image segmentation process is performed by the Attention-UNet structure to achieve high-performance segmentation results in conjunctiva images with motion blur. Motion correction processes with two steps—registration and template matching—are used to correct for large displacements and fine movements. The image displacement values decrease to 4–7 μm during registration (first step) and less than 1 μm during template matching (second step). With the corrected images, the blood flow velocity is calculated for selected vessels considering temporal signal variances and vessel lengths. These methods for resolving motion artifacts contribute insights into studies quantifying the hemodynamics of the conjunctiva, as well as other tissues.

## 1. Introduction

The conjunctiva is a translucent and highly vascularized membrane covering the sclera of the human eye. These properties enable the conjunctiva to be the only tissue observing red blood cell (RBC) shift that can be utilized for measuring the blood flow velocity directly from the surface. Quantitative analysis of the blood flow velocity has been used to estimate the progression of eye diseases, including diabetic retinopathy [1] and dry eye syndrome [2,3,4]. The diabetic retinopathy patients group had slower blood flow velocities in the conjunctiva than the control group [1]. In the case of dry eye syndrome, the normal group had slow blood flow velocities in the conjunctiva [2,3,4]. Moreover, patients with unilateral ischemic stroke [5] and high cardiovascular disease risk [6] tend to have slower conjunctival blood flow velocities. These studies demonstrated that quantifying the conjunctival blood flow velocity can contribute to evaluate not only ophthalmic diseases but, also, systemic diseases in critical organs, especially the brain and cardiovascular system.

Conventional methods for quantifying the conjunctival blood flow velocity use functional slit-lamp biomicroscopy [7], a noninvasive optical imaging system (EyeFlow) [8], and orthogonal polarization spectral imaging [9]. These methods can be disturbed by motion artifacts inherited from the image acquisition process due to eye movements. The motion artifacts cause two distinct problems: (1) image displacement and (2) low-quality images.

First, the image displacement problem causes the vessel to be misaligned from the central point. Registration was performed by calculating the difference of the correlation coefficients from the reference frame [10,11,12]. In another method, the sharpness index of each image was measured by calculating pixel-to-pixel intensity variance, eliminating the inadequate frames below the threshold value [13]. These two methods can compensate for rapid eye movements but have difficulty correcting for fibrillation or respiratory eye movements.

Second, the segmentation, which is the essential step for quantifying the microcirculation, remains challenging for low-quality images of blurry structures and uneven light illumination by subject motions. Various segmentation methods [7,10,14,15] were applied to the conjunctiva images with motion artifacts. The Frangi filter [10,14] is the most commonly used segmentation algorithm and exploits multiscale information from the eigenvalues of the Hessian matrix. The supervised method [16], which uses the Gabor wavelet filter and the Gaussian mixture model (GMM) classifier, was suggested for conjunctiva vessel segmentation [7,15]. These two segmentation methods are efficient in identifying vessels but lack of the ability to identify low-quality vessels.

We solved the image displacement and low-quality image problems caused by motion artifacts by proposing a custom-built optical system with a two-step calibration method and a deep learning-based segmentation model. The custom-built optical system was optimized to acquire human bulbar conjunctival images. The two-step calibration method was motivated by the fact that image displacements can result from sudden eye movements and respiratory movements. The first step, registration, corrects the sudden eye movements. The second step, template matching, eliminates the respiratory movements. Since deep learning-based segmentation is effective with low-quality conjunctival images [17], a custom-built Attention-UNet model was constructed to extract accurate conjunctiva vascular information. The blood flow velocity was measured by generating a spatial–temporal analysis (STA) image from the corrected image sequence and vascular features. With this configured system, we can acquire a conjunctival vascular image set with minimal motion and accurately quantify conjunctival blood flow velocity.

## 2. Materials and Methods

### 2.1. Process of Quantifying Blood Flow Velocity

**Figure 1:**
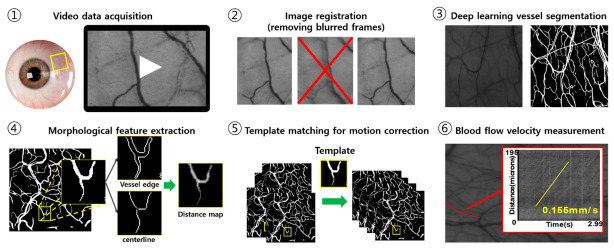
The summary of experimental phase in this study.

Quantification of the blood flow velocity is performed by the six steps shown in Figure 1. After acquiring image frames for 3 s with 25 fps, image processing, including image registration, feature extraction, and motion correction, provides motion-free image sequences for measuring the blood flow velocity through tracking the position of red blood cells. Detailed explanations of imaging acquisition, registration, and deep learning-based image segmentation and quantification are shown in following sections.

### 2.2. Image Acquisition

**Figure 2:**
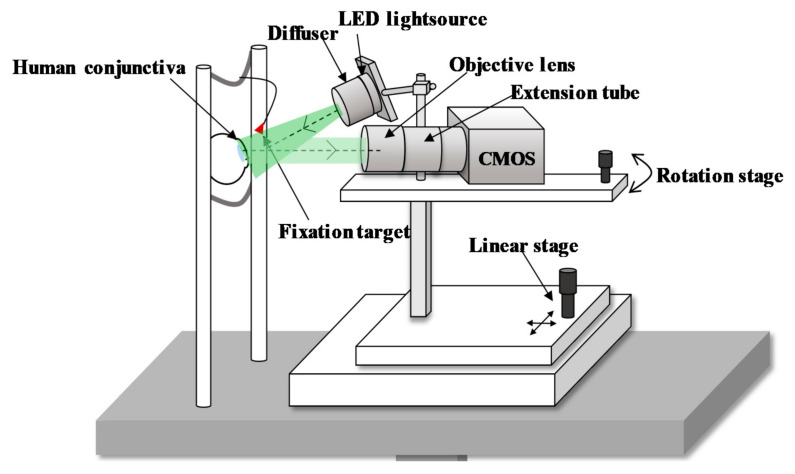
Custom-built optical imaging system for human bulbar conjunctiva.

The schematic of a customized optical imaging system is depicted in Figure 2. The conjunctival imaging system uses a green LED with a central wavelength of 525 nm and a spectral bandwidth of 5 nm, because hemoglobin and deoxyhemoglobin have high extinction coefficients at a wavelength of 530 nm. Accordingly, the image contrast between the blood vessels and the white sclera can be improved. We illuminate the uniform light using a diffuser (ED1-C50-MD, Thorlabs Inc., Newton, NJ, USA) forward to the LED. The power of the LED at the eye pupil is 300 μW/cm^2^, which is 0.3 times the laser safety standards (ANSI) limits under the condition of 10-min exposure [18].

The diffusely reflected light from the conjunctiva transmits to the complementary metal oxide semiconductor (CMOS) sensor-based camera (UI-3080CP Rev.2, IDS Inc., Obersulm, Germany) with an imaging sensor size of 8.473 mm × 7.086 mm to acquire a maximum resolution of 2456 × 2054 pixels. The pixel size on the camera sensor is 3.45 μm × 3.45 μm. The frame rate is set at 20 fps but is enhanced to 25 fps by binning the image size to 2208 × 1848 pixels for a more continuous blood flow assessment. The video data are recorded for approximately 3 s with 25 fps.

The magnification of the system is designed to achieve RBC flow imaging. An RBC with an average diameter of 7.5 μm [19] should be imaged by at least 2 pixels on the camera sensor to distinguish the individual RBC particles [20]. Moreover, the magnification for the reliable quantification of RBC flow velocity requires 4 to 5 pixels imaged per RBC [21], corresponding to 2× in our system. To achieve this magnification, we use a high-magnification zoom lens (MVL6 × 12Z, Thorlabs Inc., Newton, NJ, USA) with an adjustable magnification between 0.7× and 6×. An extension tube (MVL20A, Thorlabs Inc., Newton, NJ, USA) with 2× magnification is connected for additional magnification, for a total range of 1.832× to 7.5×. An optimized magnification is set at 3.798× for a field of view of 2.00 mm × 1.68 mm, thereby sampling each RBC with 8.26 pixels.

### 2.3. Image Registration

Image registration is the process of eliminating the blurred frames caused by rapid eye motion or blinking. Image sequences are first examined with an image contrast index that can determine the quality of the images. To obtain the contrast index, we apply the Sobel edge algorithm [22,23], a method of quantitatively measuring the contrast of an image [24]. The contrast index is calculated with the Equation (1).

(1) $$Contrast\;Index=\left(\overset{N}{\underset{y=1}{\sum }}\overset{M}{\underset{x=1}{\sum }}I_{xy}\right)/\left(M\cdot N\right)$$

where M, N are the dimensions of the image, x, y are the pixel indices of each axis, and $I_{xy}$ is the image pixel intensity. The overall image contrast is estimated by the average value of the edge intensity. The blurred frames with low-contrast indices are resolved by extracting only frames with a contrast index greater than 95% of the maximum value. A template frame is then designated based on the highest contrast index. The rest of the consecutive frames are automatically aligned to the template frame using the ImageJ plugin called motion corrector [25]. This algorithm corrects the image translation by maximizing the overlapping region between two images, thereby eliminating the significant displacement caused by rapid eye motions.

### 2.4. Deep Learning Vessel Segmentation

#### 2.4.1. Dataset

A conjunctival vessel dataset and a high-resolution fundus (HRF) dataset are used to train and evaluate the deep learning model [26]. The HRF dataset has been established by the research team at the Friedrich-Alexander Universität and used to test the effectiveness of the deep learning-based segmentation algorithm [26]. The conjunctival vessel data are collected from the conjunctiva of five healthy human subjects (five males, age = 27 ± 1) with the custom-built imaging system. This dataset contains 15 conjunctiva images with a size of 2208 × 1848 pixels. The conjunctiva images used for network learning are randomly selected in the frames extracted from image sequences without motion-blurred images. The HRF dataset comprises 45 color fundus images, equally distributed into three subsets (healthy, diabetic retinopathy, and glaucoma). Each image in the HRF dataset is 3304 × 2236 pixels. Both datasets have annotated vessel structures in the form of binary images.

#### 2.4.2. Image Preprocessing and Preparation

**Figure 3:**
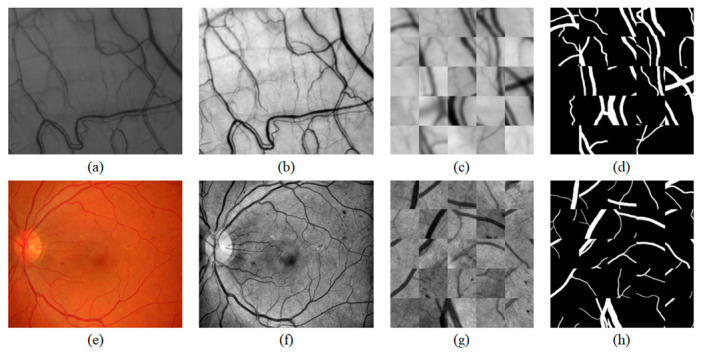
Preparation steps of the conjunctival dataset and the HRF dataset. The conjunctival data preparation step: (**a**) raw data of the conjunctival image, (**b**) CLAHE-adopted image, (**c**) conjunctiva patches, and (**d**) corresponding ground-truth of (**c**). The HRF data preparation step: (**e**) resized HRF image, (**f**) CLAHE-adopted image, (**g**) HRF patches, and the (**h**) corresponding ground-truth of (**g**).

Preprocessing enhances the contrast of the vessel in the image and removes uneven illuminations that occurred in the image acquisition step. We apply three preprocessing steps. In the first step, we crop the HRF images from the center point to the same size as the conjunctival images and resize both to 1104 × 924 pixels (0.5×). Figure 3a,e illustrates the raw data of the conjunctival image and resized HRF image. In the second step, we extract the green channel from the HRF images. The green channel has a higher contrast and lower background noise than the other channels. Finally, contrast-limited adaptive histogram equalization (CLAHE) [27] is applied in the green channel of the HRF in Figure 3f and conjunctival images in Figure 3b to enhance the contrast of the images. After preprocessing, two datasets are combined into a single dataset to enhance the generalization ability of the model.

A convolutional neural network (CNN) requires large amounts of training data to prevent overfitting of the network and improve the generalization ability. To train the dataset, we exploit a patch-wise strategy [17,28,29,30] and data augmentation. The patch-wise strategy is used to learn a small amount of data efficiently and overcome the memory limitations caused by high-image resolution. This strategy randomly extracts patches in the range of 64 to 128 pixels. The patch sizes from 65 × 65 pixels to 128 × 128 pixels are resized to 64 × 64-pixel patches. After resizing the extracted patches, overlapped regions of the conjunctiva in each different-sized patch are recognized as different regions in the network model.

A total of 300,000 patches are obtained by sampling 5000 patches from each image. Figure 3c,g are examples of patch-wise extractions. Figure 3d,h are the corresponding ground-truth images of the patches (Figure 3c,g) for the supervised learning of the convolutional neural network.

Data augmentation is applied to extract the patches with additional vascular features to improve the CNN generalization ability. We applied data augmentations such as geometrical distortions (rotation, shearing, and transformation) and motion blur. Geometrical distortions can increase the representation of the patches. Motion blur is used to learn the deformed vessel based on the movements that occurred in the image acquisition step. The patches are normalized to the zero mean and unit variance before the training process to reduce the effect of the large intensity variance.

#### 2.4.3. Network Architecture

**Figure 4:**
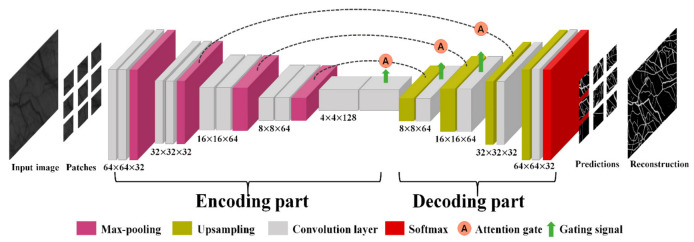
Customized Attention-UNet architecture.

The Attention-UNet architecture [31] is adopted to learn the vascular features. We customize the Attention-UNet to optimize our datasets. The details of the architecture are described in Figure 4. The architecture is based on a layered CNN, consisting of an encoder–decoder structure with three stages and an attention mechanism.

The encoder gradually reduces the spatial dimension of the input to learn a low-resolution feature map. Each stage of the encoder consists of two convolution layers and one max-pooling layer. At the end of the encoder stage, a bottom layer exists without max-pooling. Whenever the stage progresses to the next stage, the filter size of the convolution layer doubles, and the dimensions of the input are halved. Each convolution layer comprises a 3 × 3 convolution filter with a stride of 1, batch normalization, and a rectified linear unit (ReLU).

The decoder enables precise localization by merging the low-resolution features from the previous layer and high-resolution features from the encoder of the same stage. When the low-resolution features are transported, the upsampling process, which is implemented by transposing a convolution kernel (kernel size = 3 × 3, stride = 2), reconstructs the salient features from the input. Before the encoder transfers the features, an attention gate is used to suppress the irrelevant background of the input and highlight the relevant foreground features. At the end of the 3-stage decoding, the last convolutional kernel (kernel size = 1 × 1) and SoftMax activation function are used for mapping the feature vector and classify the vessel.

#### 2.4.4. Model Training and Testing

The deep learning model using Keras is trained and validated on a CPU (Xeon(R) silver 4112, Intel Corporation, Santa Clara, CA, USA) and a GPU (Quadro P4000, Nvidia Corporation, Santa Clara, CA, USA) operated by Ubuntu (16.04 LTS, Canonical Ltd, London, UK).

In the training process, the complete set of augmented patches is split into 240,000 for training the network and 60,000 for validation. The training process has 150 epochs with the strategy of reducing the learning rate on the plateau. A validation set is used to evaluate the performance of the model in each epoch. If the performance of the model in the validation set does not change in 15 epochs, the strategy will reduce the learning rate by 1/10. The training of the model is progressed by an adaptive moment estimation (Adam) optimizer (initial learning rate = 0.00005) and the Dice coefficient [32] as the loss function.

In conjunctival images, blood vessel information occupies a small portion of the entire image compared to the background region. Therefore, the Dice coefficient is used to solve the class imbalance problem. The Dice coefficient is defined in the Equation (2):

(2) $$Dice\;Coefficient=\frac{2\sum_{i}^{N}p_{i}\cdot q_{i}}{\sum_{I}^{N}p_{i}{}^{2}+\sum_{i}^{N}q_{i}{}^{2}}$$

where $p_{i}$ is predicted segmentation map, and $q_{i}$ is the binary ground-truth image. N denotes the number of pixels in each image, and i is the position of the pixel in the image.

In the test phase, the CNN infers the test image, excluding the training dataset. The test image is generated by averaging 30 frames to distinguish the obscure vessel from the registered conjunctival images. By inferencing the test data using the optimal model for validation, a reference segmentation map is acquired.

### 2.5. Morphological Feature Extraction

The vessel length and diameter are measured from segmented conjunctival vessel images. Distinguishing the connected vessel segments is necessary to extract these morphological vessel features. The centerline and intersection points of the vessels are required to separate individual vessel segments. The centerline is obtained by a skeleton image using the pixel-wise thinning algorithm [33,34], a method of performing an iterative process until it remains one pixel wide in the segmented vessels. Skeleton segments lower than 20 pixels are removed, because these segments are not recognized as a connected vascular network. The intersection points at bifurcation and crossover are determined by the number of neighbors, a convolution result with a 3 × 3 unity kernel for each pixel of the centerline. The bifurcation points correspond to three in the convolution result, and the crossovers have a result greater than three. By removing these two points, each vessel segment is separated and given identification. We measure the vessel length and the diameter from the identified vessel segment. The length of the vessel is obtained by counting each pixel of the skeletonized vessel along its centerline. Moreover, the vessel diameters are measured in Euclidean distance by calculating the perpendicular distance from the centerline to the nearest background of the binary segmented vessels.

### 2.6. Template Matching for Motion Correction

The template matching algorithm is used for correcting the fine movements in image sequences caused by respiratory movement. First, the template image is assigned by selecting a template vessel considering the morphological features, including the vessel length and diameter. The template vessel must be contained in all frames and distinguished from other blood vessels. Equation (3) is applied to each vessel segment to select a vessel of the template image with a long length and large diameter.

(3) $$N\left(L,D\right)=w_{1}\cdot L+w_{2}\cdot D$$

$N\left(L,D\right)$ is the function for selecting the template vessel, $w_{1},w_{2}$ are the weight factors, L is the length of the vessel segment, and D is the diameter of the vessel. The vessel length and diameter are normalized to equalize the scales of each parameter. The vessel segment with the highest value of the function is determined as the template vessel.

We generate the template image by cropping the selected template vessel to the minimum bounding box. The template-matching algorithm based on the assigned template image is applied to the target frames, and this algorithm is implemented with the cvMatchTemplate function in the OpenCV library [35]. This function calculates the normalized correlation coefficient $R\left(x,y\right)$ at each pixel to search the most similar region with the template image, as shown in the Equation (4):

(4) $$R\left(x,y\right)=\frac{\sum_{x’,y’}(T\left(x’,y’\right)\cdot I\left(x+x’,y+y’\right))}{\sqrt{\sum_{x’,y’}T{\left(x’,y’\right)}^{2}\cdot \sum_{x’,y’}I{\left(x+x’,y+y’\right)}^{2}}}$$

where T is the template image, I is a source image to find a match with the template image, x,y is the pixel location of the source image, and x’,y’ indicates the pixel location of the template image. After finding the most similar region to the template image with the source images, the displacement value is obtained from the center point of the source image. We shift the source images as much as the displacement value, thereby successfully correcting the fine movements.

### 2.7. Blood Flow Velocity Measurements

Several blood vessels can be observed in motion-corrected conjunctival images, but a RBC shift is not detectable in all blood vessels. Measuring the blood flow velocity requires distinguishing the blood vessels capable of detecting the RBC shift. Generally, vessels with measurable blood flow have high temporal variance in the centerline due to the RBC shifts. Moreover, vessels with longer lengths are more conducive to measuring the blood flow velocity, because the movements of RBC can be observed continuously for a long duration. Considering the temporal variance and vessel length, an index of observability is defined as shown in the Equation (5):

(5) $$Observability\;Index=\alpha \cdot \sigma_{t}+\beta \cdot L$$

where α,β is the weighting factor, $\sigma_{t}$ is the temporal variance, and L is the length of the vessel segments. Vessels with a high index are considered to be capable of measuring the blood flow velocity. We choose 15 vessel segments with the highest observability index to analyze the blood flow velocity.

**Figure 5:**
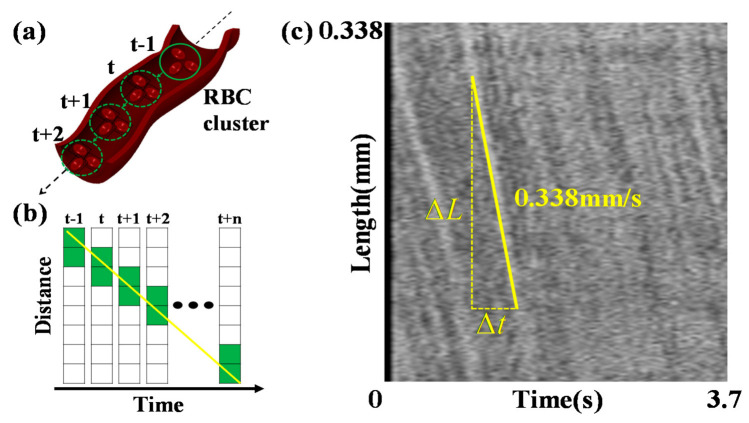
(**a**) The RBC cluster shifts over time in the vessel centerline. (**b**) Pixel intensity of the vessel centerline changes due to the RBC cluster shift. (**c**) Spatial–temporal analysis (STA) image generated by the pixel intensity from the vessel centerline as stacking at each column. The x and y axes indicate the frame time and vessel length, respectively. The yellow line shown in the STA image displays the slope, indicating the blood flow velocity.

The blood flow velocity is measured by tracking the RBC movements in the selected vessels centerline, as depicted in Figure 5a. Tracking is performed using the spatial–temporal analysis (STA) method, which demonstrates an alteration of the pixel intensity of the centerline due to the RBC movements. Figure 5b displays an example of an alteration in the pixel intensity of the vessel centerlines as a function of time. We generate the STA image by stacking the centerlines to each column, as depicted in Figure 5c. Frames corresponding to 3.7 s are stacked to form 70 columns. Consequently, the flow of the RBC cluster forms lines consistent with the yellow line in Figure 5c, with the slope on the STA image. The blood flow velocities are measured by calculating the average values of the slopes.

## 3. Results

### 3.1. Segmentation

**Figure 6:**
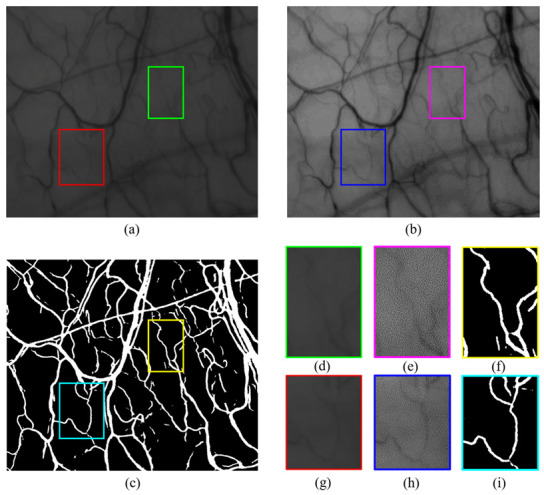
(**a**) Averaged conjunctival image. (**b**) Brightness and contrast-adjusted (**a**) by Image J (set display range: 25–115). (**c**) Attention-UNet segmentation results. (**d**,**g**) Cropped images from the low-contrast, blurry areas of (**a**). (**e**,**h**) Cropped images from (**b**). (**f**,**i**) Corresponding to the prediction results of (**d**,**g**). Brightness and contrast-adjusted images (**b**,**e**,**h**) were placed to provide easier visibility for the reader.

We identified blurry, low-contrast conjunctival vessels by constructing a dataset mixed with conjunctiva images and HRF to train the custom-built Attention-UNet model. The segmentation map was obtained using model prediction on the averaged image. Figure 6 illustrates the results of the model prediction. The conjunctival image of Figure 6a is unseen data obtained from healthy subjects. Figure 6b,e,h are additional processed images to show the unseen data of Figure 6a,d,g for readers. Figure 6c results from the model prediction in Figure 6a. Each box with a color boundary in Figure 6a–c represents regions of interest for the low-contrast, blurry vessels. These results demonstrate that Attention-UNet trained with mixed datasets is accurate for low-contrast vascular structures without additional postprocessing.

### 3.2. Motion Correction

**Figure 7:**
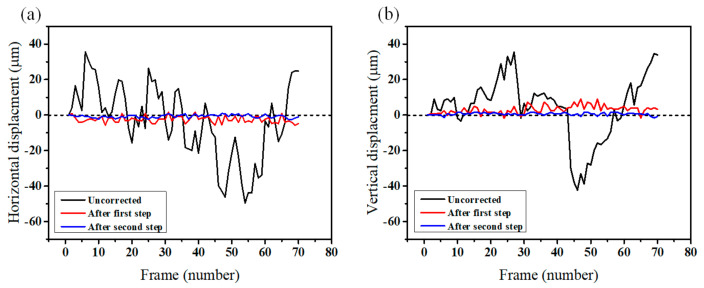
Comparison of the displacement values between uncorrected (black line) and motion-corrected (red and blue lines) image sequence. Red line indicates the displacement values after the first registration step, and the blue line represents after the second motion correction step. (**a**) Horizontal displacement values. (**b**) Vertical displacement values.

To evaluate the performance of the motion correction processes, we compare the displacement values of 70 frames of the conjunctival microvessels. Figure 7 illustrates the horizontal and vertical axial displacement values of the source images from the first frame. In the uncorrected case (black line), the intense axial motions of the frames are visible. After the correction process, the axial motions are noticeably reduced (red and blue lines). For the horizontal axis depicted in Figure 7a, the mean axial displacement decreased to 2.69 μm from 16.84 μm after the first registration process. After the second process of motion correction, it decreased to 0.9 μm. For the vertical axis depicted in Figure 7b, it also decreased to 0.81 μm from 14 μm. Consequently, most of the displacement values decreased, except for the movements smaller than 1 μm.

**Figure 8:**
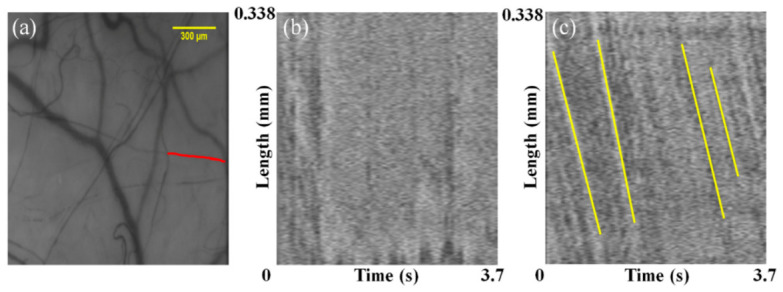
(**a**) Vessel used to generate the STA image (red line). (**b**) STA image before motion correction. (**c**) STA image after motion correction. Yellow lines represent slopes, which indicate blood flow velocity.

Furthermore, we compared before and after the motion correction of the spatial–temporal analysis (STA) images, which are crucial to quantifying the blood flow velocity. The red line in Figure 8a displays a target vessel to analyze the blood flow velocity. Figure 8b illustrates an STA image before motion correction. In this STA image, the slope required to calculate the blood flow velocity cannot be verified because of the motion artifacts. In contrast, the clear edges of the slope displayed by the yellow line in Figure 8c are observed in the STA image after motion correction. Finally, the blood flow velocity obtained from the average values of the yellow slopes is 0.338 mm/s.

Table 1 illustrates the characteristics of conjunctival microvessels, including diameter, length, and blood flow velocity. These characteristics are measured in the selected vessel segments with the highest observability indices. Starting with V1, 10 blood vessels with a high observability index are sequentially arranged. The minimum and Table 1 illustrate the characteristics of the conjunctival microvessels, including diameter, length, and blood flow velocity. These characteristics are measured in the selected vessel segments with the highest observability indices. Starting with V1, 10 blood vessels with a high observability index are sequentially arranged. The minimum and maximum blood vessel diameters are 8.172 and 15.62 μm. The blood flow velocity ranges between 0.078 and 0.338 mm/s, similar to the values in a previous study, were measured with other equipment [36].

## 4. Discussion

In this paper, we introduced a system that can accurately quantify the conjunctival blood flow velocity by overcoming motion artifacts. First, Attention-UNet was implemented to precisely segment the low-quality vessel images. The Attention-UNet trained with a retinal dataset was used to segment conjunctival vessels with low-contrast, blurry structures [17]. This study inferred that Attention-UNet has a high generalization ability to learn the vascular structure.

Second, we conducted a two-step correction process to solve the problem of changing local information. Fine movements are critical to high magnification imaging to track red blood cells (RBC) for measuring the blood flow. Although we corrected a large motion through the registration process, 4–7 μm of the displacement remained. An additional correction process was essential to obtain an accurate blood flow velocity by tracking RBC particles of approximately 7.5 μm in diameter [19]. Therefore, we implemented an additional motion correction algorithm, template matching, by considering the vessel features, including diameter and length. The displacements of the conjunctival microvasculature images are reduced to the order of 1 μm while minimizing the frame loss.

We construct a custom-built optical system to image the human conjunctiva and acquire the conjunctival images from five healthy subjects. Conjunctival datasets have a risk of overfitting due to a lack of images, which we avoided by adding a retinal dataset with a similar domain to the conjunctival images. The high-resolution fundus (HRF) dataset was selected as an additional dataset because of the vessel size similar to our conjunctival image. The model trained by the mixed dataset achieved more accurate segmentation results than the conjunctival dataset only.

Furthermore, our motion correction process can produce insights in observing blood flow velocity for an extended period by correcting their fine control movements. When the human eye gazes at a fixed object, the dwelling time ranges from 90 to 900 ms [37]. After the dwelling time, the fixated eyes start vibrating. Due to eye movements caused by the short dwelling time, conjunctival hemodynamics were observed for only 0.3 s in a previous study [10].

However, the velocity pulse period (VPP), which is the time varying the blood flow velocity, due to the cardiac impulse is 940 ms [38], longer than the dwelling time. Consequently, it is necessary to observe the blood flow velocity for a more extended period than the VPP. Since we compensate for the motion above the VPP, the blood flow velocity is quantified above three seconds through the STA image. We created an opportunity for quantifying the long-term blood flow assessment, limited by a dwelling time shorter than the cardiac cycle time.

A limitation in the current configuration is that it can be difficult to compensate for the motion blur caused by movements that are faster than the frame rates. This type of image can be blurred, even if the location is not changed. One way to mitigate this problem is to reduce the exposure time and increase the frame rate. However, such an approach would inevitably decrease the contrast of the image. We overcame this limitation by comparing the contrast index assigned during registration, thereby removing the blurred frames with low-contrast values.

This study adopted several capabilities, including image registration, deep learning vessel segmentation, and template matching for motion correction, to quantify the microcirculation of the human conjunctiva. Using these methods, we acquired a blood flow velocity of 0.078 to 0.338 mm/s in the conjunctiva vessels. Although we could not perfectly control the factors affecting the blood flow velocity, we could confirm that our results partially corresponded to a previous study measuring the conjunctiva blood flow range as 0.19 to 0.33 mm/s [36].

As further works, our image processing method could provide blood flow velocity in the retina, wrists, lips, and fingernails. In addition, when significant correlations of conjunctival hemodynamics with cardiovascular diseases, as well as diabetes, are demonstrated, the developed imaging system and processing method can be used as one of the methods providing pre-diagnostic factors for systemic diseases [1,39].

## 5. Conclusions

We demonstrate a system that resolves motion artifacts to quantify the conjunctival blood flow velocity. Deep learning-based segmentation and motion correction techniques are used to solve the motion artifacts during image acquisition. We evaluated the system performance by analyzing conjunctival images from five healthy volunteers. The system segment low-contrast vessels reduced the image displacement to less than 1 to 2 μm. Pathways of red blood cells could be tracked free from the motion artifacts, resulting in quantifying the blood flow velocity. The range of quantifying the conjunctival blood flow velocity is 0.078~0.338 mm/s in a healthy subject. This conjunctival imaging instrument is applicable for imaging subjects with limited forward-looking capabilities or an unsteady fixation.

## Figures and Tables

**Table 1:** Diameter, length, and blood flow velocity of conjunctival microvessels.

Vessel	Diameter (μm)	Length (mm)	Blood Flow Velocity (mm/s)
V1	13.158	0.414	0.086
V2	15.282	0.356	0.097
V3	8.172	0.338	0.338
V4	9.878	0.330	0.090
V5	10.170	0.318	0.270
V6	8.682	0.220	0.141
V7	9.574	0.250	0.078
V8	15.422	0.246	0.137
V9	15.620	0.128	0.114
V10	9.934	0.214	0.153

## Data Availability

In this paper, both publicly available datasets and custom datasets were used. Publicly available datasets were analyzed in this study. This data can be found here: https://www5.cs.fau.de/research/data/fundus-images/, accessed on 3 May 2021. The custom data presented in this study are available on request from the corresponding author. The data are not publicly available due to privacy.
